# The influence of benzene on the composition, diversity and performance of the anodic bacterial community in glucose-fed microbial fuel cells

**DOI:** 10.3389/fmicb.2024.1384463

**Published:** 2024-07-15

**Authors:** Natalia Tyszkiewicz, Jaak Truu, Piotr Młynarz, Grzegorz Pasternak

**Affiliations:** ^1^Laboratory of Microbial Electrochemical Systems, Department of Process Engineering and Technology of Polymer and Carbon Materials, Faculty of Chemistry, Wrocław University of Science and Technology, Wrocław, Poland; ^2^Department of Biochemistry, Molecular Biology and Biotechnology, Faculty of Chemistry, Wrocław University of Science and Technology, Wrocław, Poland; ^3^Institute of Molecular and Cell Biology, University of Tartu, Tartu, Estonia

**Keywords:** MFC, petroleum, biodegradation, microbial biodiversity, metabolomics

## Abstract

Bioelectrochemical systems offer unique opportunities to remove recalcitrant environmental pollutants in a net positive energy process, although it remains challenging because of the toxic character of such compounds. In this study, microbial fuel cell (MFC) technology was applied to investigate the benzene degradation process for more than 160 days, where glucose was used as a co-metabolite and a control. We have applied an inoculation strategy that led to the development of 10 individual microbial communities. The electrochemical dynamics of MFC efficiency was observed, along with their ^1^H NMR metabolic fingerprints and analysis of the microbial community. The highest power density of 120 mW/m^2^ was recorded in the final period of the experiment when benzene/glucose was used as fuel. This is the highest value reported in a benzene/co-substrate system. Metabolite analysis confirmed the full removal of benzene, while the dominance of fermentation products indicated the strong occurrence of non-electrogenic reactions. Based on 16S rRNA gene amplicon sequencing, bacterial community analysis revealed several petroleum-degrading microorganisms, electroactive species and biosurfactant producers. The dominant species were recognised as *Citrobacter freundii* and *Arcobacter faecis*. Strong, positive impact of the presence of benzene on the alpha diversity was recorded, underlining the high complexity of the bioelectrochemically supported degradation of petroleum compounds. This study reveals the importance of supporting the bioelectrochemical degradation process with auxiliary substrates and inoculation strategies that allow the communities to reach sufficient diversity to improve the power output and degradation efficiency in MFCs beyond the previously known limits. This study, for the first time, provides an outlook on the syntrophic activity of biosurfactant producers and petroleum degraders towards the efficient removal and conversion of recalcitrant hydrophobic compounds into electricity in MFCs.

## 1 Introduction

Benzene is a common environmental pollutant that occurs frequently in the soil, air and aquatic environments. It is classified as an aromatic hydrocarbon, and due to its highly stable nature, its toxicity, and low water solubility, it is difficult for microorganisms to degrade (Varjani et al., 2017). The main source of contamination with this compound is the petroleum industry: for example, petroleum refining, fuel combustion in vehicles, polymer production and leaks of petroleum products from storage tanks and pipelines. Its presence in the environment has a negative impact on plants, animals and human health, making this compound one of the main environmental concerns with hundreds of thousands of identified pollution sites only in the EU (JRC, 2018).

Several classic biodegradation methods have been developed over the last decades that comprise *in situ* and *ex situ* methods such as bioaugmentation or bioremediation in prisms. These techniques generally require the use of additional electron acceptors, such as oxygen or nitrates, to perform and complete the degradation processes (Liu et al., 2018). Furthermore, they are often combined with ventilation, which can cause contaminants released into the air (Alori et al., 2022). Another disadvantage is the electrical energy consumption when using such techniques. Therefore, it is essential to search for new methods for the removal and neutralisation of benzene in the environment.

These drawbacks can be overcome through the development of new technologies, such as bioelectrochemical systems (BES) technology. The best-known example of such technology is microbial fuel cell (MFC). The key elements of these systems include membranes, electrodes and biotic elements, which can provide a wide variety of functions (Edel et al., 2022). Electroactive species convert organic compounds into electrical energy through respiratory processes. In recent years, MFCs have gained significant attention due to their possible application in the removal of contaminants with simultaneous energy production (Suresh et al., 2022). When microorganisms have a stable, constant electron sink, it is possible to generate electricity, while omitting the need to supply other, costly electron acceptors.

However, there are several challenges in implementing the MFC technology in polluted sites. Because benzene is a recalcitrant compound, it is often required to apply co-metabolites to allow or increase the efficiency of the biodegradation. Such co-metabolic biodegradation usually depends on compounds such as sugars, volatile fatty acids, or alcohols (Shen et al., 2020). These co-metabolites were typically required in most of the reported studies, where benzene was the target for biodegradation (Table 1). Glucose is one of the most commonly used co-substrates in MFCs. Its presence could also increase the bioavailability of hydrophobic substrates for microorganisms and affect the increasing removal efficiency (Obileke et al., 2021). Some of the few exceptions were reported by Liu et al., who investigated benzene degradation during contaminated groundwater treatment in tubular MFCs. They have recorded a maximum power density of 3.9 mW/m^2^ (Liu et al., 2018). In another study, Adelaja et al. (2018) recorded a power output of 0.82 mW/m^2^ when investigating the biodegradation process supported by the addition of glucose.

**Table 1:** MFC performance and comparison with other studies used aromatic hydrocarbons as substrates.

Type of inoculum	Carbon source	Type of MFC	Max power density [mW/m^2^]	Max power density [W/m^3^]	COD removal [%]	CE [%]	References
**Complex microbial community**	**0.6 g/L benzene and 1.2 g/L glucose**	**sc-MFC**	**119.62**	5.38	**80.43**	8.4	**This study**
Complex microbial community petroleum hydrocarbon acclimated	0.06 g/L benzene/groundwater	sc-MFC	3.9	N.A.	N.A.	N.A.	Liu et al. (2018)
Benzene and ammonium-contaminated groundwater	0.05 g/L benzene/groundwater	sc-MFC	N.A.	0.316	N.A.	14	Wei et al. (2015)
Coculture of *S. oneidensis* and *P. aeruginosa*	0.2 g/L benzene, 0.1 g/L glucose or pyruvate	dc-MFC	0.39	N.A.	61.76	0.36	Adelaja et al. (2018)
Adapted anaerobic digested sludge			0.82	N.A.	87.25	1.04	
Anaerobic digested sludge with coculture of *S. oneidensis* and *P. aeruginosa*			0.14	N.A.	70.0	0.2	
Mixed microbial community petroleum hydrocarbon acclimated	1.5 g/L benzene, 0.1 g/L phenanthrene	sc-MFC	6.75	N.A.	77	N.A.	Adelaja et al. (2017)
Mixed bacterial community from the oil-cracking wastewater treatment plant	0.1 g/L benzene	dc-MFC	0.0205	N.A.	N.A.	N.A.	Wu et al. (2013)
Mix of aerobic and anaerobic activated sludge	1 g/L phenol	dc-MFC	N.A.	9.1	N.A.	1.5	Luo et al. (2009)
	0.5 g/L glucose, 1 g/L phenol	dc-MFC	N.A.	28.3	N.A.	2.7	
Anaerobic activated sludge	0.3 g/L phenanthrene, 0.2 g/L benzene	dc-MFC	1.06	N.A.	79.1	0.4	Adelaja et al. (2015)

sc-MFC, single-chamber MFC; dc-MFC, dual chamber MFC; N.A., not available.

Performing the anaerobic benzene degradation process, coupled with anode respiration pathways, is a challenging task. Most likely, this was the principal reason that none of the reported studies have demonstrated the ability of a single bacterial culture to carry out this process in MFCs. A study by Zhang et al. reported using a pure culture of *Geobacter metallireducens* in the removal of benzene but in hydrocarbon-contaminated sediments (Zhang et al., 2010). It contains several thousands of other compounds, which affect benzene biodegradation pathways and overall MFC performance. Power performance values reported in literature have never exceeded 6.75 mW/m^2^ (Adelaja et al., 2017) and 12.7 mW/m^2^ in multi-stacked MFCs (3.9 mW/m^2^ for a single unit) (Liu et al., 2018). Therefore, the recorded power outputs were very low compared to simple substrates such as acetate (de Rosset et al., 2023) but also other hydrocarbons such as phenol (Luo et al., 2009).

In recent years, there has been an ongoing discussion of the optimal diversity of microbial communities. Several authors have concluded that diversity is strongly related to the overall environmental complexity of the system in which biodegradation is performed. In a recent study, the authors investigated the long-term adaptation of activated sludge, which was under continuous stress from the presence of benzene and naphthalene (Li et al., 2022). The authors reported an ongoing downward trend in microbial diversity during long-term operation, although a higher abundance of petroleum degraders was detected. This effect is related to the fact that most microorganisms are not tolerant to the increased presence of harmful chemicals (Wang et al., 2023). A recent study by Zhuang et al. revealed that soil microbial diversity decreases when exposed to high levels of petroleum compounds C10–C40 (Zhuang et al., 2023). The authors observed increased levels of diversity when higher concentrations of petroleum compounds were present, which is in contrast to the findings of Peng et al. (2022), who indicated that under elevated levels of oil refining waste, diversity was severely restricted, while few dominant genera still contributed to petroleum degradation. When the above complexity of interactions between microbial communities and environmental parameters, in particular, in bioelectrochemical systems aimed for biodegradation is taken into account, it is therefore crucial to understand the microbial growth, diversity and metabolism of benzene in microbial fuel cells. Understanding the mechanisms of this process is required to fully implement the bioelectrochemical system technology in field applications.

The purpose of this study was to investigate the efficiency of individual microbial species isolated from MFC communities, as well as complex communities derived from activated sludge in benzene degradation. We determined the impact of benzene and glucose on the structure and metabolism of the microbial community to understand the influence of these two substrates on the development of efficient consortia abundant in petroleum degraders, biosurfactant producers and electroactive species. Developing various communities and investigating their structure and efficiency are important for the practical implementation of MFC technology in environmental biodegradation.

## 2 Materials and methods

### 2.1 MFC configuration

Single-chamber air-cathode MFCs, manufactured using 3D-printing (polypropylene, Fiberlogy, Poland), were used (Supplementary Figure S1). The anode electrode with a geometric area of 9 cm^2^ was made up of carbon veil 30 g/m^2^ (PRF Composite Materials, Dorset, United Kingdom). The cathode consisted of carbon paste (carbon CWZ-22 mixed with water) and a stainless-steel mesh current collector. Both anode and cathode were connected by Ni-Cr wire (Ø 0.66 mm, ALBu, Poland) to the resistor. The electrodes were separated by a cation exchange membrane (CMI-7000, Membranes International, United States). The displacement volume was 20 mL. Prior to inoculation, MFCs were disinfected with 70% (v/v) ethanol solution. Then, each of the MFCs was washed with sterile water three times to remove excessive alcohol.

### 2.2 MFC inoculation

For inoculation, initially five different bacterial strains isolated from soils contaminated with petroleum compounds were used and identified as *Ochrobactrum anthropi* strain 1 (MFC 1), *Rhodococcus qingshengii* (MFC 2), *Epilithonimonas hominis* (MFC 3), *Novosphingobium lubricantis* (MFC 4), and *Ochrobactrum anthropi* strain 2 (MFC 5). The inoculates were suspended in mineral salt medium (MSM) prepared according to Pasternak et al. (2023) with carbon source (series A—13.7 mM of glycerol, series B—10.7 mM of benzene) to obtain the same final density of 1.0 on the McFarland scale. Due to the low power density values, after 18 days of operation, the carbon substrates were replaced with glucose (6.7 mM, 1.2 g/L) in series A and benzene along with glucose (benzene: 10.5 mM, 0.6 g/L; and glucose: 6.7 mM, 1.2 g/L) for series B. After 40 days of operation, MFCs were supplemented with 10 μL (0.05% (v/v)) of activated sludge, which was collected from the local wastewater treatment plant (Ścinawka Dolna, Poland). By adding a minimal quantity to each MFC, we aimed to develop a minimal complexity consortium capable of supporting the current generation, alongside pure strains isolated from contaminated environments.

### 2.3 MFC operation

In total, the set of 10 MFCs was operated in fed-batch mode with a 3-day replacement frequency. The MFCs operated at constant room temperature (25°C) at the same time. The MFCs were divided into two groups, denoted as A and B, as mentioned in detail in the previous paragraph. At the beginning of the experiment, each of the resistor was set up to 2.2 kΩ. After 6 days of MFC operation, the resistors were changed to 2.0 kΩ and then adjusted to the values calculated from the polarisation experiments.

### 2.4 MFC performance

#### 2.4.1 Real-time temporal performance

The MFC voltage was monitored over a period of 160 days. Data were collected using BenchVue Keysight DAQ970A Data Acquisition System (Keysight Technologies, United States). The voltage generated by the MFCs was measured in real-time mode and recorded every 3 min. Power densities were calculated according to Ohms law as follows:


$$\mathrm{Power}\;\mathrm{density}=\frac{U^{2}}{R*A}\;\left[\frac{W}{m^{2}}\right]\text{,}$$

where *U* is the voltage [V], *R* is the resistance [Ω], and *A* is the geometric surface area of the anode electrode [m^2^].

#### 2.4.2 Linear sweep voltammetry

Two- and three-electrode polarisation experiments were carried out. In both setups, an anode electrode was used as the working electrode, and a cathode electrode was used as the counter electrode with saturated Ag/AgCl as the reference electrode. The linear sweep voltammetry (LSV) experiments were carried out using multichannel potentiostat (VSP, BioLogic). The programme was set from open circuit potential (OCP) to 0 V with a scan rate of 1 mV/s. All data were normalised to the geometric surface area of the anode electrode.

#### 2.4.3 Chemical oxygen demand and Coulombic efficiency

Anolyte samples were collected from MFCs at the end of the feeding batch cycle and then centrifuged (6 min, 6,000 r.p.m.). The supernatant was filtered and used to determine the chemical oxygen demand (COD) using the COD kit (HACH, Unites States).

Coulombic efficiency (CE) was determined by integrating the current measured over time (t), compared with the theoretical current on the basis of COD removal, and calculated as the next equation:


$$C_{E}=\frac{\mathrm{Coulombs}\;\mathrm{recovered}}{\mathrm{Total}\;\mathrm{coulombs}\;\mathrm{in}\;\mathrm{substrate}}$$

$$C_{E}=\frac{8\int_{0}^{t_{b}}I\;dt}{Fv_{\mathrm{An}}\Delta \mathrm{COD}}$$

where 8 is a constant value used for COD, *F* is a Faraday’s constant, $v_{\mathrm{An}}$ is a volume of the anolyte inside the anode chamber, and ΔCOD is the change in COD concentration during a batch cycle.

### 2.5 Metabolomic analysis

#### 2.5.1 Sample preparation

The samples were collected from MFCs at the end of the feeding batch cycle. For the determination of extracellular metabolites, each anolyte was centrifuged for 6 min at 6,000 r.p.m. and then the supernatant was filtered through a sterile syringe filter (0.22 μm). The samples were stored at −20°C before analysis. A sample volume of 2 mL was introduced into Eppendorf tubes and evaporated in a vacuum centrifuge at 45°C for 6 h (Eppendorf, Poland) and dissolved in 600 μL of PBS buffer (0.1 M NaH_2_PO_4_/Na_2_HPO_4_, 10% D_2_O, pH = 7.0, TSP = 0.3 mM). Subsequently, 550 μL was transferred to 5-mm NMR tubes (5SP, Armar Chemicals, Germany) for measurements.

#### 2.5.2 ^1^H NMR studies

Parameter settings were as previously described in the study by Mielko et al. (2021). One dimensional ^1^H NMR experiments were performed on a Bruker AVANCE II spectrometer (Bruker, GmBH, Germany) equipped with a 5-mm TBO probe at 298 K and 600.58 MHz. Spectra were carried out using the cpmgpr1d pulse sequence by suppression of water resonance by presaturation. The spectra were referenced to the TSP resonance at 0.0 ppm and manually corrected for phase and baseline (MestReNova v. 11.0.3, Qingdao, China).

### 2.6 Bacterial community structure analysis

#### 2.6.1 DNA extraction

Samples for DNA isolation were collected from the anode chambers on the 96 days of the experiment. Total DNA was extracted using the Bead-Beat Micro AX Gravity kit manufactured by A&A BIOTECHNOLOGY (Warszawa, Poland). The quality and quantity of the DNA extracts were evaluated using spectrophotometric measurements performed with the NanoDrop^™^ 2000/2000c instrument from Thermo Fisher Scientific (United States).

#### 2.6.2 16S rRNA gene sequencing

The nucleotide sequences are deposited in a public database at the accession DOI number: https://doi.org/10.18150/IHWMJF. The DNA isolated from pure cultures was analysed by sequencing approximately 1,000 bp using BigDye^®^ Terminator v3.1 kits, then separated using the 3730xl DNA Analyser and matched against the BLAST database. Bacterial community taxonomic profiling was conducted through Illumina^®^ MiSeq sequencing. PCR amplification and library preparation utilised universal primers 341F and 785R (Klindworth et al., 2013). The Q5 Hot Start High-Fidelity 2X Master Mix from New England BioLabs was used as the reagent kit in PCR reactions, following the recommended conditions provided by the manufacturer. Paired-end sequencing was carried out on the MiSeq device using the Illumina v3 kit, resulting in read lengths of 2x300nt. Bioinformatics analysis was performed using the QIIME 2 platform and DADA2 package. The initial bioinformatics analysis included quality control and sequence preprocessing. Quality control involved the generation of dynamic control parameters based on the error model specific to each sample using the FIGARO tool. Furthermore, quality control was performed to ensure that the maximum expected error rates of each sample were met. The sequences obtained were pre-processed using the Cutadapt tool. Taxonomic classification of the representative sequences of amplicon sequence variants (ASVs) was performed using the Silva 138 reference database with a two-step hybrid method. First, an exact match of reads with the reference database was performed using vsearch. Any reads that were not recognised in the previous step were classified using a machine learning-based method (sklearn) available in the QIIME q2-feature-classifier module (Bokulich et al., 2018).

#### 2.6.3 Statistical analysis

Principal component analysis (PCA) and heatmaps were produced using the R packages FactoMineR and pheatmap. Centred log ratio transformation was applied to relative abundance values before performing PCA and clustering. Differences in bacterial community structure between MFCs with and without benzene addition were assessed with permutational multivariate analysis of variance (PERMANOVA) with 9,999 permutations using the Bray–Curtis dissimilarity matrix, implemented in the MicrobiomeAnalyst software (Chong et al., 2020). The paired *t*-test was applied to compare the values of the bacterial community abundance and diversity estimate between MFCs with and without the addition of benzene.

#### 2.6.4 Quantitative PCR

Quantitative polymerase chain reaction was used to assess gene copy number. QPCR was carried out using the Rotor-Gene^®^ Q real-time PCR system (Qiagen, Foster City, CA, United States). Analysis was performed as previously described by Tiirik et al. (2021) using A519F and A915R as primers. Then, 10-fold dilutions of DNA samples were used for the amplification process. Detailed characterisation of primers and programme is included in Supplementary Table S1.

## 3 Results and discussion

### 3.1 Power performance

The power generation was recorded in real time for more than 160 days. At the beginning of the experiment, when glycerol (A) and benzene (B) were used as fuel, only low power density of <5 mW/m^2^ was recorded. Subsequently, when the substrates were changed on day 18 to glucose (A) and benzene and glucose (B), the power generated by the MFCs increased several times. However, the introduction of new substrates has resulted in only relatively low power output, up to 12 mW/m^2^ for (A) and 2 mW/m^2^ for (B). These weak results suggest very limited capabilities of individual strains for power production and a toxic effect of benzene, by inhibiting the metabolic activities of the microorganisms and decreasing their abundances.

**Figure 1:**
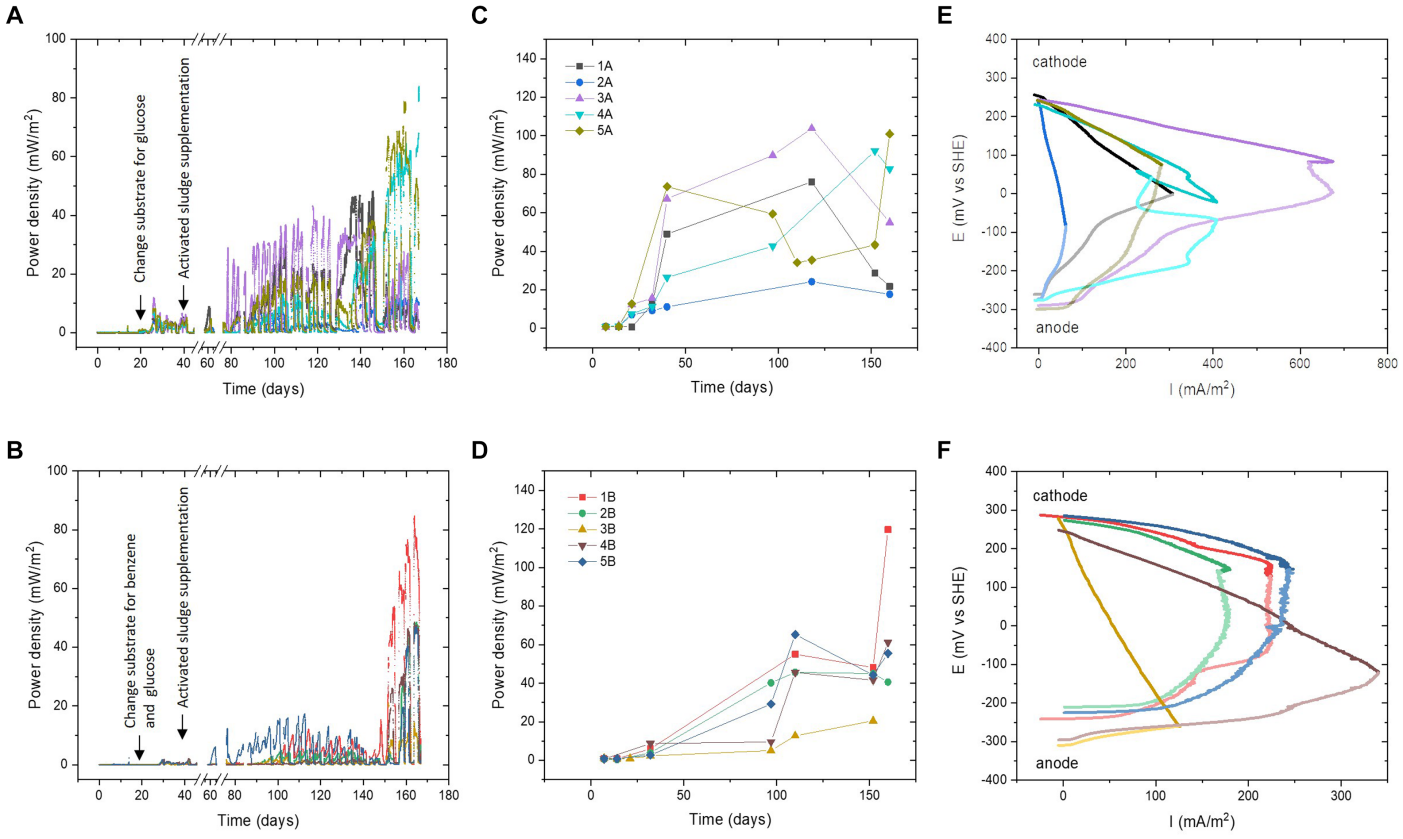
Real-time temporal power performance **(A,B)**. Maximum power density values from LSV tests **(C,D)**. Polarisation curves of the anodes and cathodes of the MFCs **(E,F)** at maximum performance (1A, 2A, 3A, 5A, 2B, and 5B—120th day; 4A, 1B, 3B, and 4B—150th day); the colours for the anode potentials are less intense. The colours in the legend of panels **(C,D)** are valid for all panels in the same row. Panels **(A,C,E)** are series A, and **(B,D,F)** are series B.

The addition of activated sludge was carried out after 40 days of the experiment to facilitate benzene degradation. Real-time power performance monitoring (Figures 1A,B) revealed a significant increase in power density starting from the 60th day of operation. When individual MFCs are compared, it is clear that the presence of benzene has elongated the adaptation time needed for the microbial communities to reach their maximum power performance. Among all MFCs, the fastest adaptation to the efficient microbial consortium was recorded in MFC 3A, initially inoculated with *Epilithonimonas hominis*, where the power output increased by 30 mW/m^2^ after approximately 40 days of activated sludge supplementation. On the contrary, for MFC 3B, where benzene was used along with glucose, a significant increase in power density was recorded at the end of the experimental period, after 150 days.

Different behaviours were observed for MFC 1A, initially inoculated with *Ochrobactrum anthropi* strain 1. It revealed a stable increase in power over time, reaching a maximum of 40 mW/m^2^ after 140 days. Adapting the same starting consortium with benzene resulted in a higher power output (MFC 1B), equal to 85 mW/m^2^, which was the highest power density among all MFCs, reaching levels similar to those of MFCs that were fed without benzene (series A). MFC 5A and MFC 5B (initially *Ochrobactrum anthropi* strain 2) achieved similar real-time power behaviour throughout the experiment. However, after 140 days, higher power generation was recorded for the cell without benzene. The maximum power density values for MFC 5A and MFC 5B were approximately 65 and 45 mW/m^2^, respectively. MFC 2A and MFC 2B (initially *Rhodococcus qingshengii*) reached similar power until day 150, where MFC 2B supplemented with benzene was more efficient and reached a value of 45 mW/m^2^ vs. 15 mW/m^2^ for the MFC 2A. Overall, it may be possible that the adaptation time to reach a fully matured biofilm and stable community was higher due to the initial presence of pure cultures on the anodic surface. It is known that anodic biofilm structure is very stable and hostile against external bacterial strains once the biofilm has matured (Ieropoulos et al., 2019). Thus, the new consortium established over the anodes required more time to adaptation and resulted in peak performance after 140 days of operation.

The open circuit voltage (OCV) ranged from 0.49 V to 0.59 V for glucose-fed MFCs. The highest value was recorded for MFC 3A, which exhibited the highest power density value in the experiment. For MFCs supplemented with benzene and glucose, OCV ranged from 0.51 V to 0.54 V, with the highest value for MFC 2B. The graphs of the maximum power densities calculated from the polarisation tests (Figures 1C,D) show that MFC-fed glucose reached higher power generation values and were more stable over time. Benzene-supplemented MFCs showed an increasing trend throughout the duration of the experiment. At the end of the experimental period, these MFCs have reached similar power density values as MFCs fed with glucose. In the benzene group, the highest power density value of the LSV tests was 120 mW/m^2^ (108 μW) for MFC 1B, and the lowest was 21 mW/m^2^ (18 μW) for MFC 3B. In the glucose-fed group, the highest power value was 104 mW/m^2^ (93 μW) for MFC 3A, and the lowest was MFC 2A with a power density of 24 mW/m^2^ (22 μW).

Figures 1A,B clearly suggested that the dynamic community evolution that took place in glucose-fed MFCs was more divergent and led to higher variation of the results over time. On the other hand, the presence of benzene resulted in stabilising the electrical signal over time and led to less divergent long-term data, suggesting higher similarities between the compositions of these communities. This was further confirmed using 16S rRNA biodiversity analysis.

The results achieved in this study showed that the co-metabolic degradation of benzene was significantly higher than showed by other studies (Table 1). Power output was almost 100 times higher compared to the study reported by Adelaja et al. (2018), where the maximum power density was 0.82 mW/m^2^. The authors have used a dual chamber MFC design, along with a 3-fold lower benzene concentration and a 12-fold lower glucose concentration. However, in this study, MFCs with pure cultures in the first 18 days of operation achieved power performance of approximately 4.5 mW/m^2^. We also identify these values as negligible power, even for pure cultures MFCs.

These results indicate the importance of the presence of co-metabolite in MFC system to degrade petroleum compounds such as benzene. Microorganisms exposed to benzene took a relatively long time to adapt to harsh environmental conditions (Lovley, 2000). Furthermore, when individual MFCs were compared, long-term power performance was similar when benzene was added as a substrate, while much higher variability of power output was observed for glucose as a sole source of carbon.

The performance of the individual electrodes was investigated at the end of the experimental period (as indicated in Figures 1E,F). The polarisation curves obtained for series A and B differ significantly in their characteristics, which mean that different factors were limiting their performance. In series A (glucose), strong ohmic losses were observed for MFC 1A and MFC 2A with dominance of the anodic overpotential. The performance of MFC 3A, MFC 4A, and MFC 5A was mainly limited by anodic concentration losses (mass transfer effect), resulting in a power overshoot phenomenon (MFC 3A and 4A). This could have been caused by the biofilm overgrowth and thus lower substrate transfer into the deeper layers of the biofilm. It is also possible that in the initial period, when negligible performance was observed for isolated species, this resulted in contamination of the electrodes with inactive cells (Greenman et al., 2021). A strong overshoot phenomenon was observed for both the anode and the cathode. Although in the cathode this could be associated with difficulties in oxygen delivery to active electrode sites and disruption of electron flow, due to salt precipitation on the cathode surface (de Rosset et al., 2023), strong underperformance of the anodes could also have affected cathodic behaviour.

Analysis of the polarisation curves of MFCs supplemented with benzene (Figure 1F) indicates that MFC 1B, MFC 2B, and MFC 5B were limited by ohmic resistance and concentration losses at the anode. A primary reason for this behaviour in benzene-degrading MFCs could be the complexity and bioavailability of this substrate (Pasternak et al., 2022). For MFC 3B and MFC 4B, losses were observed in all regions of the cathodic curves. Apart from the salt accumulation, the cathodes can undergo biofouling, which can negatively impact their durability (Pasternak et al., 2016).

### 3.2 Coulombic efficiency and COD removal

**Figure 2:**
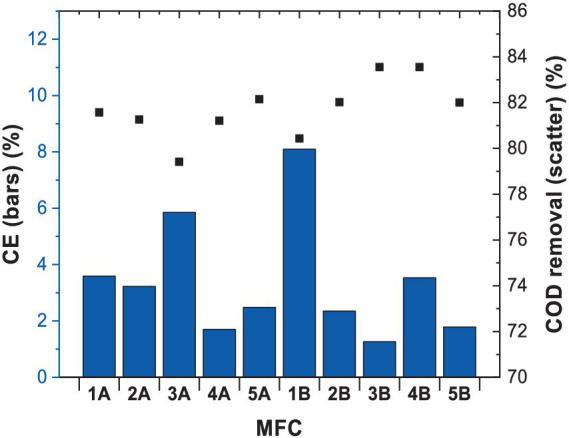
CE and COD values measured after 3 months of MFCs operation.

Coulombic efficiency (CE) refers to the efficiency with which electrons produced during microbial processes are transferred to the electrode as electrical current. In our study (Figure 2), CE values ranged from 1 to 8%, indicating that electrochemical reactions were responsible for a minor part of total COD removal. However, CE values were higher when benzene and glucose were used together as carbon sources in MFCs. The highest CE values observed were of 8.4% vs. 5.9% when glucose was used as a single substrate. The higher CE values for the combination of benzene and glucose may be due to the fact that the microbial population that oxidises those substrates differs from each other. This contributes to the release of electrons when attacking the respective substrates and hence to the higher values obtained in comparison with those obtained for MFC using only glucose. This could have resulted from various metabolic fluxes represented by different microbial consortia, in particular the presence of fermentative pathways that compete with anodic respiration (Torres et al., 2007).

However, data from other studies (Table 1) indicate that CE achieved in this study, when benzene was used as fuel (8.4%), was the highest reported so far. Adelaja et al. (2017) carried out a similar study, where anaerobic digested sludge was used as an inoculum to degrade benzene with glucose as a co-metabolite, resulting in CE of 1.04%. The use of coculture of *Shewanella oneidensis* and *Pseudomonas aeruginosa* resulted in CE of 0.36%. Luo et al. (2009) used phenol as a fuel, leading to a CE of 1.5%, which improved significantly when glucose was added to facilitate degradation (CE = 2.7%). In another study by Adelaja et al. (2015), phenanthrene and glucose degradation were characterised by CE of 0.4%.

Herein, the observed CE was one of the highest values reported in the literature for benzene in MFC. In general, glucose results in lower CE values, compared to low-molecular-weight compounds such as acetate, because of its fermentative nature. In addition to electroactive species, glucose could be used by non-electrogenic bacteria such as methanogens and fermentative species (Chae et al., 2009). Glucose, however, is a common co-substrate that can be used in conventional biodegradation studies (Liu et al., 2023), which was confirmed herein for bioelectrochemical degradation, through a significant improvement in Coulombic efficiency.

Nevertheless, the high removal of COD achieved of 80–90% still indicates that a significant portion of glucose and benzene was metabolised in a non-electrogenic manner. Metabolite analysis through NMR studies allowed us to provide insight into the potential metabolic pathways involved and led to the decrease in the current generation. Similarly as with CE and maximum power output, the COD values reported herein were among the highest compared to other studies in which this parameter ranged from 26.3 to 87.3%.

### 3.3 Metabolites

**Figure 3:**
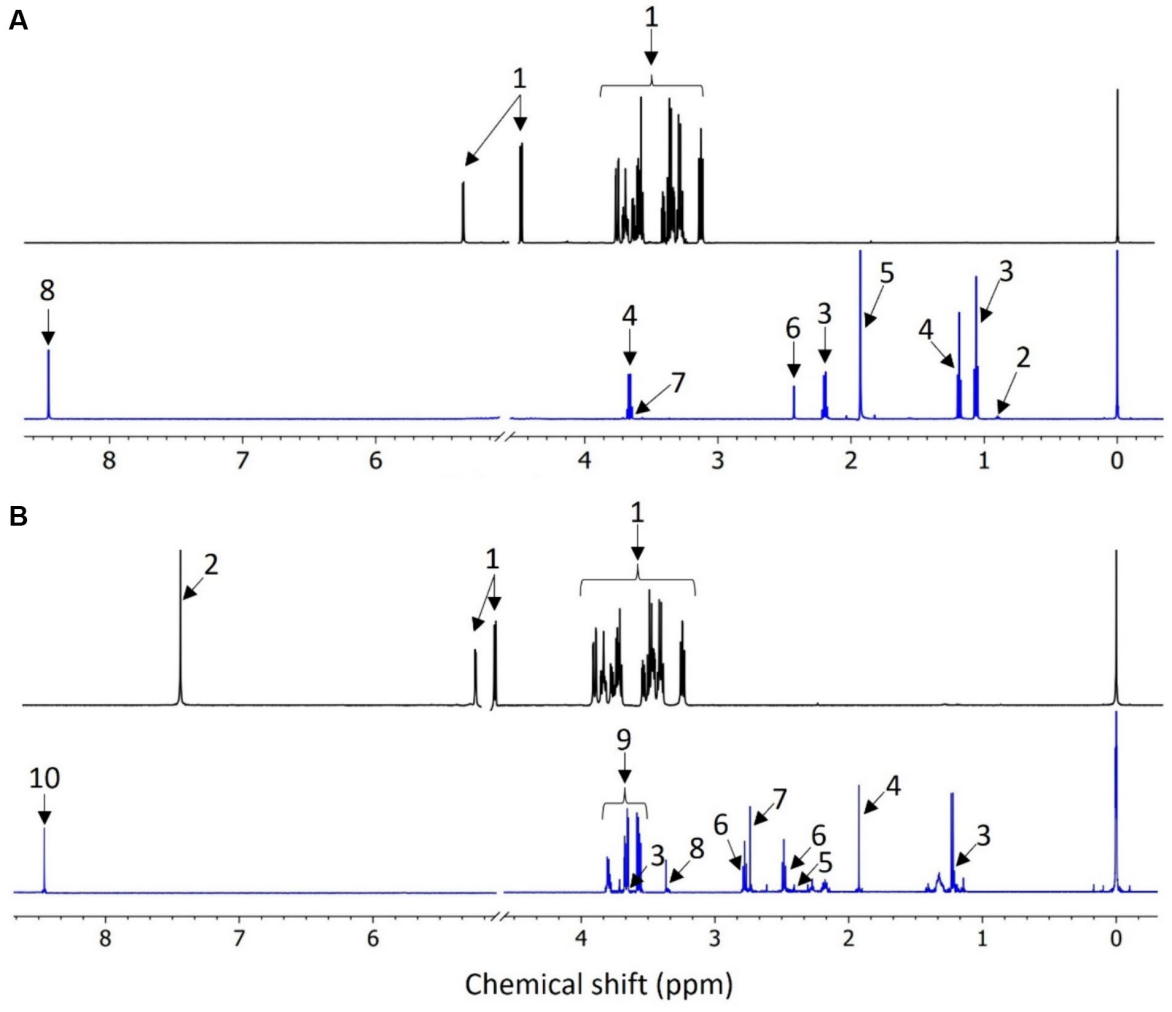
Comparison of 1D ^1^H NMR cpmgpr1d spectra of **(A)** MSM medium with glucose (top) and the representative sample obtained from MFC 1A (bottom) (1: glucose; 2: butyrate; 3: propionate; 4: ethanol; 5: acetate; 6: succinate; 7: glycerol; 8: formate); **(B)** medium with benzene and glucose (top) and the representative sample obtained from MFC 4B (bottom) (1: glucose; 2: benzene; 3: ethanol; 4: acetate; 5: succinate; 6: 5-aminolevulinate; 7: dimethylamine; 8: methanol; 9: glycerol; 10: formate).

The metabolic analysis for group A fed with glucose (Figure 3A) revealed seven distinct exometabolites (released to the electrolyte), while for group B fed with benzene and glucose (Figure 3B), eight metabolites were present. Five of the metabolites were common for both groups: ethanol, acetate, succinate, glycerol and formate (see Table 2). The presence of these compounds indicates fermentation reactions that include acetogenesis, which occurs under anaerobic conditions.

**Table 2:** ^1^H NMR signal assignments for extracellular metabolites in MFCs.

L.p.	Metabolites	KEGG number	Chemical shift of representative signal [ppm]	
			Group A	Group B
1	Ethanol	C00469	3.65 (m)	1.20 (t)
2	Acetate	C00033	1.91 (s)	1.91 (s)
3	5-aminolevulinate	C00430	–	2.47 (m)
4	Succinate	C00042	2.39 (s)	2.39 (s)
5	Dimethylamine	C00534	–	2.72 (s)
6	Methanol	C00132	–	3.35 (s)
7	Glycerol	C00116	3.55 (dd)	3.65(dd)
8	Formate	C00058	8.44 (s)	8.44 (s)
9	Butyrate	C00246	1.54 (m)	–
10	Propionate	C00163	1.04 (t)	–

Identified metabolites are products that can be delivered from both fermentative glucose reactions and microaerophilic benzene transformation (Toth et al., 2021). The oxygen concentration in the anodic chambers was measured below 1% vs. O_2_ in the air, indicating that benzene degradation could occur under microaerophilic and anaerobic conditions. This initial oxygen content may have caused aerobic degradation to predominate at the beginning of the feeding batch cycle and succeeded under anaerobic conditions, once oxygen was depleted.

Some of the identified metabolites, such as acetate, formate and propionate, are known to be end products of both aerobic and anaerobic metabolic pathways of benzene degradation (Firmino et al., 2018). The presence of these carboxylic acids may also indicate the appearance of methanogens, which was further confirmed by 16S rRNA analysis. Bioelectrochemical degradation did not lead to the presence of any metabolites, typical for the aerobic and anaerobic degradation of benzene, such as catechol and benzoate, which were not identified. This is due to the fact that the sampling was carried out at the end of the feeding batch cycle, where the final products of the metabolic pathways are the most abundant.

Under methanogenic conditions, aromatic hydrocarbons are degraded by syntrophic interactions between fermentative bacteria and methanogenic archaea, rather than by a single microbial species. Numerous studies have explained this phenomenon, in which fermentative bacteria convert aromatic hydrocarbons to intermediate metabolites such as acetate and propionate. These metabolites serve as substrates for methanogenesis by archaea (Vogt et al., 2011).

Under aerobic conditions, benzene is converted into catechol via cis-benzene dihydrodiol and then attacked through ortho- or meta-ring cleavage. The resulting product is pyruvate, which can then be converted to formate, acetate, or ethanol (Liu, 2003) as detected here. Acetate could be the final product of benzene anaerobic degradation under sulphate-reducing conditions, which can occur in MFCs, and through methylation, hydroxylation and carboxylation, converted to benzoyl-CoA as the central metabolite that can be further reduced by benzoyl-CoA reductases (Vogt et al., 2011). Low CE values are caused by the competition for the carbon source between electroactive and other microorganisms, mainly methanogenic bacteria, which are known to convert benzene into methane (Grbic-Galic and Vogel, 1987).

### 3.4 Microbial community analysis

**Figure 4:**
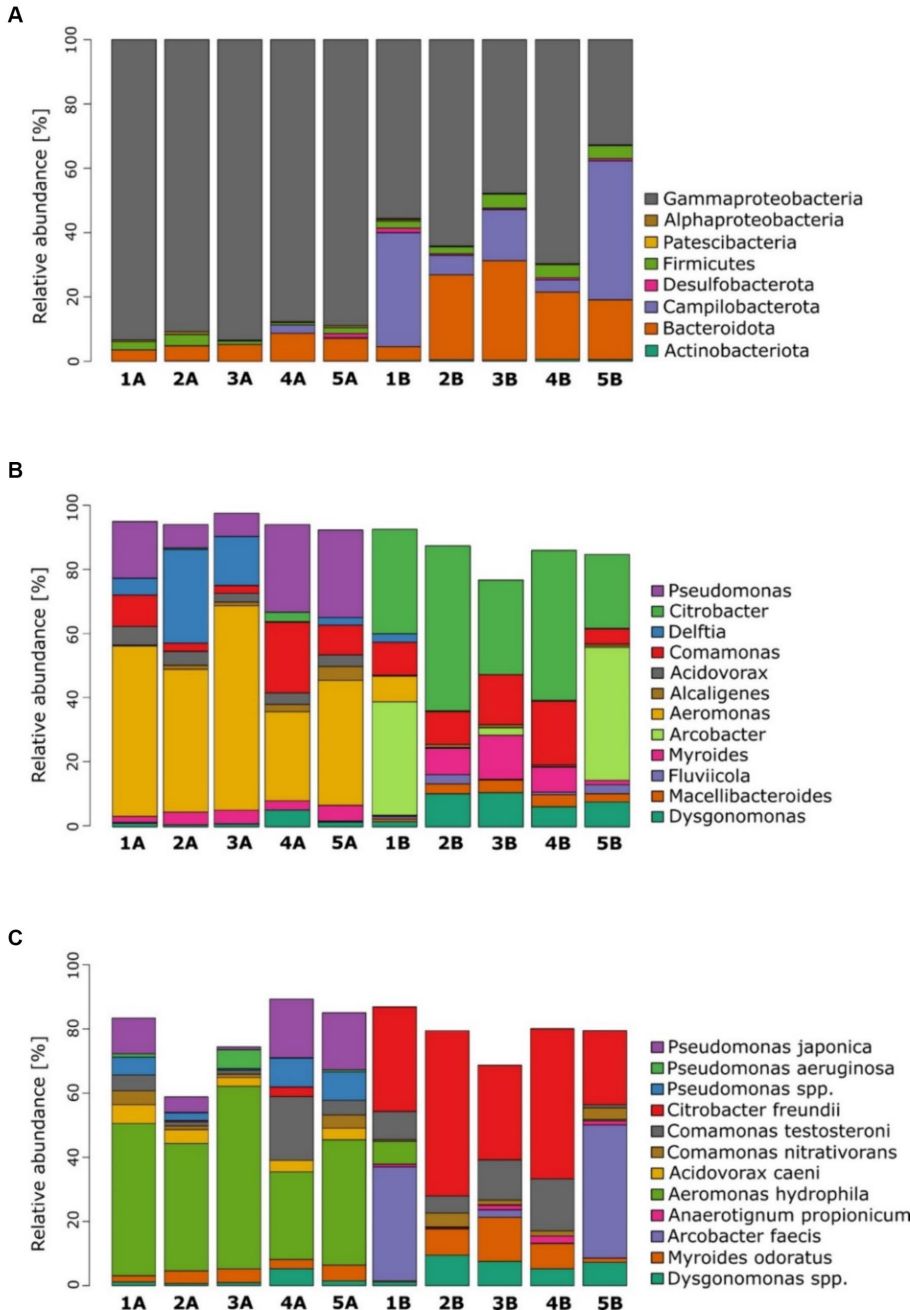
Microbial community analysis for anodic communities fed with glucose (group A) and glucose and benzene (group B). Plots **(A–C)** show abundance profiling by the most abundant phyla, genera and species, respectively.

Microbial community analysis revealed distinct microbial fingerprints. When comparing individual communities, those subjected to glucose exhibited a higher abundance of *Proteobacteria* (mainly *Gammaproteobacteria*), while MFCs supplemented with benzene demonstrated a higher abundance of *Campylobacterota*, *Bacteriota,* and *Actinobacteriota* (Figure 4A). Moreover, MFCs 3B, 4B, and 5B displayed a higher amount of *Firmicutes*.

**Figure 5:**
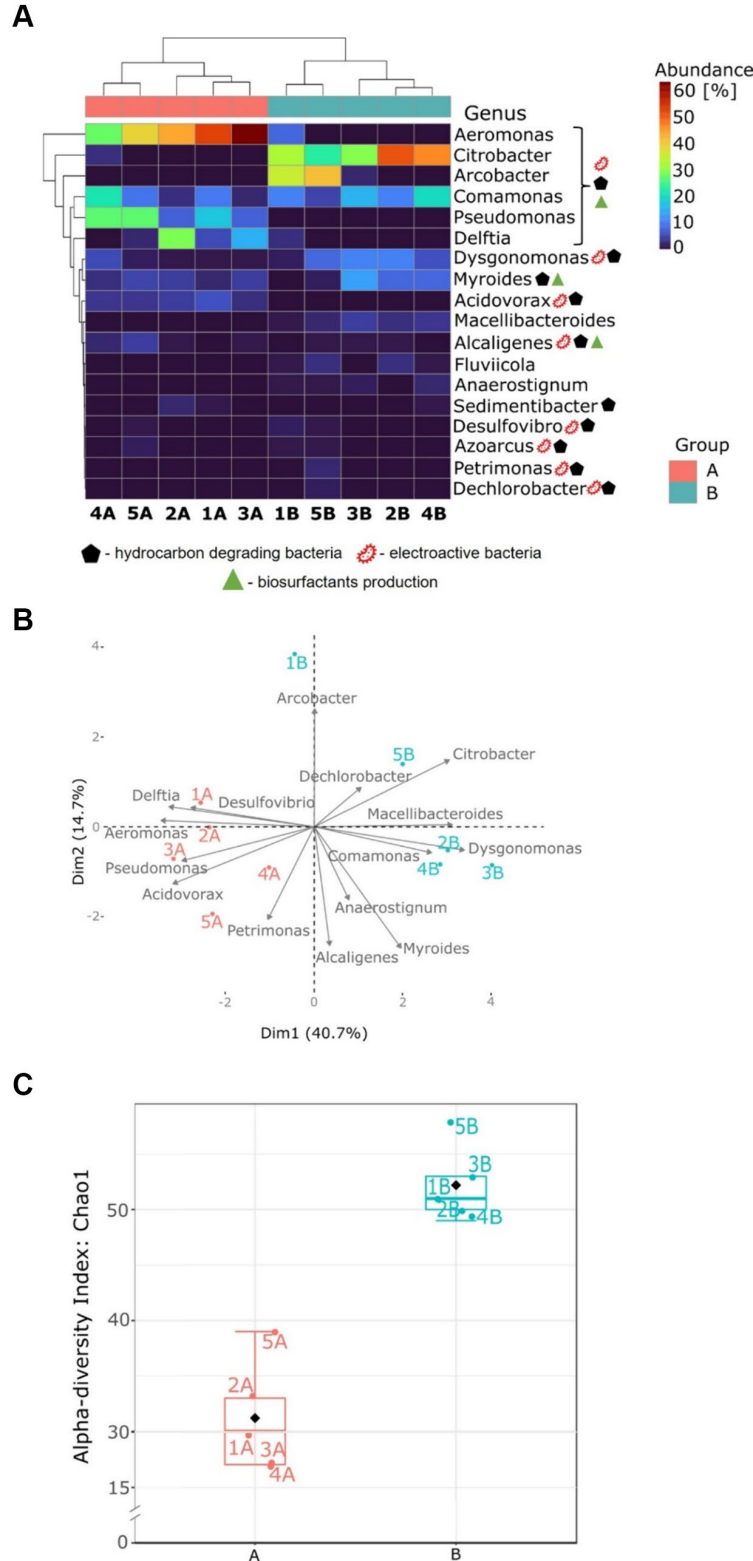
Microbial community analysis for anodic communities fed with glucose (group A) and glucose and benzene (group B). **(A)** Heatmap of the most abundant genera; **(B)** PCA plot based on the most abundant genera; **(C)** box and whiskers plots of Chao1 index at the genus level.

At the genus level (Figure 4B), microbial communities of group A were dominated mainly by *Aeromonas,* followed by *Pseudomonas* and *Acidovorax*. Figure 5A indicates the species identified as key electroactive and petroleum-degrading species, as well as biosurfactant producers. *Aeromonas* genus is a group of Gram-negative bacteria that are facultative anaerobes. They exhibit electrochemical activity (e.g., *Aeromonas hydrophila*) and the ability to reduce nitrate and sulphate using various electron donors such as glucose, glycerol, and pyruvate (Pham et al., 2003). Furthermore, MFCs 1A, 2A, and 3A were co-dominated by *Delftia,* which is recognised for its proficiency in electrochemical processes and its ability to thrive in anaerobic conditions (Jangir et al., 2016). The microbiomes exposed to benzene were more diverse, and several genera were co-dominant: *Citrobacter*, *Arcobacter*, *Dysgonomonas*, *Myroides,* and *Macellibacteroides*.

At the species level (Figure 4C), the glucose-fed MFCs were dominated by *Aeromonas hydrophila*. The greatest prevalence occurs in MFCs 3A (56.9%) and 1A (47.5%), which were characterised by the highest power output generated during the experiment. This strain exhibits electroactive properties, suggesting that it plays a key role in the current generation in these MFCs (Pham et al., 2003). The glucose-fed communities were also abundant in the *Pseudomonas* genus: *Pseudomonas japonica and Pseudomonas aeruginosa*. The presence of *P. japonica* is expected in the presence of glucose (Pungrasmi et al., 2008); nevertheless, it was not enriched when glucose and benzene were used in MFC. *P. aeruginosa* is well known to perform indirect extracellular electron transfer, due to the production of various redox mediators, such as pyocyanin (Pérez-García et al., 2023). Its highest contribution was recorded as 5.9% in the most effective MFC 3A. Similarly, *Acidovorax caeni* was previously reported in anodic communities (Li et al., 2023). Its highest contribution is 5.8% in MFC 1A. Other prominent species observed in anodic communities subjected to glucose were *Comamonas testosteroni* (the highest value of 19.9% in MFC 4A), *Comamonas nitrativorans* (4.4% in MFC 1A), *Myroides odoratus* (4.9% in MFC 5A), and *Alcaligenes faecalis* (4.3% in MFC 5A), which occur commonly in activated sludge.

The bacterial community was different when benzene was used along with glucose as a fuel. The most dominant strain was *Citrobacter freundii*, whose contribution ranged from 23.1% for MFC 5B to 51.5% for MFC 2B. It is an exoelectrogenic bacterium (Huang et al., 2015), which could be responsible for the anaerobic biodegradation of benzene (Gupta et al., 2015). The high content of this strain in MFCs may suggest its important role in electron transport to the anode. For MFC 5B and MFC 1B, the most abundant strain was *Arcobacter faecis* with a contribution of 41.5 and 35.5% contribution, respectively. To the best of our knowledge, there are no data available in the literature on the activity of this species in MFCs. However, many other species of *Arcobacter* have been defined as electroactive and form biofilms in microaerobic and anaerobic environments (Hassan et al., 2018). In the remaining MFCs, its abundance was below 2%. In further studies, these strains should be verified for their degradation capabilities, along with their possible pathogenicity and drug resistance.

*Comamonas testosteroni,* classified as electroactive strain (Yu et al., 2015), was also present in MFCs operating with benzene. Its abundance was significant for all MFCs except MFC 5B and ranged from 5.3% for MFC 2B to 16.2% for MFC 4B. It belongs to *Burkholderiales* order, which is known as a dominant phylotype in enrichment cultures of anaerobic benzene-degrading microcosms. The genera of this order use the methylation pathway for anaerobic benzene activation (Melkonian et al., 2021). Another distinctive species in the benzene-degrading consortia was *Myroides odoratus* (13.7%, MFC 3B), *Aeromonas hydrophila* (7.2%, MFC 1B), *Comamonas nitrativorans* (4.3%, MFC 2B), and *Anaerotignum propionicum* (2.2%, MFC 4B). Furthermore, all benzene-degrading consortia comprised *Alcaligenes faecalis*, *Arcobacter faecis,* and *Desulfovibrio vulgaris* (<1%).

According to previous studies, *Geobacter* was the key genus in benzene-degrading communities (Rooney-Varga et al., 1999). In fact, in our study, *Geobacter* species were only found in MFCs fed with benzene, and their presence was only below <0.2%, which suggests its supporting role in the entire community. In addition, families such as *Arcobacteraceae*, *Enterobacteriaceae*, *Dysgonomonadaceae*, *Crocinitomicaceae*, *Oscillospirales*, *Nocardiaceae* and *Rikenellaceae*, known from the degradation of petroleum compounds (Ejaz et al., 2021; Duncan et al., 2024), were observed only in community-fed benzene. Some of the representatives of these families were reported as dominant genera in MFCs with benzene supplementation (*Citrobacter and Arcobacter*). Furthermore, we have identified several biosurfactant-producing species from *Sphingobacteriaceae*, as well as anaerobic members from *Paludibacteraceae*, *Anaerovoracaceae*, and *Crocinitomicaceae* and obligate anaerobes from *Oscillospirales*. The presence of these species is crucial in anaerobic benzene degradation as biosurfactants play an important role in increasing the bioavailability of petroleum compounds (Kaczorek et al., 2018).

The PCA (Figure 5B) revealed a clear separation between groups A and B. This indicates that the addition of benzene strongly impacted the bacterial community structure in the anode chambers. In MFCs supplemented with benzene, bacterial communities have a higher proportion of *Citrobacter*, *Comamonas*, *Dysgonomonas*, *Arcobacter*, *Macellibacteroides*, and *Dechlorobacter*. It can also be seen that MFC 2B, MFC 3B, and MFC 4B form a distinct group. In glucose-fed MFCs, the microbial structure was shaped mainly by the *Aeromonas*, *Pseudomonas*, *Acidovorax*, *Delftia*, *Desulfovibrio* and *Petrimonas* genera. Therefore, the PCA unveils key microbial genera responsible for the bioelectrochemical degradation of benzene and suggests that the presence of glucose in both types of metabolism is insufficient to maintain some of the important electroactive groups (such as *Pseudomonas*) within the consortia, which may have played an important role in a long adaptation process, where low power outputs were recorded.

The alpha-diversity index (Chao1, Figure 5C) calculated at the genus level was significantly different (*t*-test, *p* < 0.01) for communities adapted to the benzene cometabolism. The highest alpha-diversity index was reached by MFC 5B, and it was 1.5 times higher than the index for MFC 5A, which had the highest diversity in group A. This indicates that the presence of toxic and complex substrates in the anode chamber led to the formation of more diverse communities, compared to those exposed to a simple carbon source. This highlights that communities abundant in glucose-metabolising species possess stronger competition mechanisms that limit their abundance. The metabolism of benzene is more complex and requires more steps to fully degrade it. Therefore, a more diversified microbial community is required to allow efficient biodegradation. Moreover, according to the qPCR results (see Supplementary Table S2), the abundance of total bacterial DNA in MFCs fed glucose and benzene was slightly higher compared to MFCs fed only glucose. For Archaea, DNA copy number was an order of magnitude higher for MFCs fed with benzene, indicating that the presence of benzene was more favourable for their growth. These results indicate that the complexity of the system in which the biodegradation is performed is an important factor to take into account when analysing the process. Previous findings led to several contradictory observations, where petroleum compounds led to decrease or increase in alpha diversity, or led to increased diversity (Li et al., 2022; Peng et al., 2022; Wang et al., 2023; Zhuang et al., 2023). The principal reason could be the fact that each study varied in methodology, as well as a variety of alpha-diversity indices have been reported. Therefore, the complexity of the dynamics of microbial consortia, along with their metabolic networks in the presence of recalcitrant compounds in bioelectrochemical systems, may vary compared to other environments. This comes from the fact that such an environment is highly dynamic and self-adapts in real time to the conditions provided by the electroactive consortium itself, for example, by changing the electrochemical parameters of the reactors (Pasternak et al., 2018).

## 4 Conclusion

In this study, we have demonstrated a significant improvement in the efficiency of bioelectrochemical benzene degradation compared to previous similar studies reported in the literature. The power output reported here was 18 times higher, compared to the other benzene/co-substrate studies, and it ranged from 21 mW/m^2^ to 120 mW/m^2^, highlighting the potential of MFCs in benzene remediation. Initial inoculation with pure strains probably affected the formation of anodic biofilms after activated sludge enrichment. Differences in MFC performance with the same substrates resulted from the differences in the abundance of electroactive microorganisms in the biofilm. These results highlight the importance of initial inoculation strategies to optimise MFC performance.

This study underscores the critical importance of dynamic adaptation of microbial consortia reaching a high diversity of electroactive microbial communities for efficient degradation of benzene in MFCs. Bacterial community analysis revealed that benzene-degrading communities were distinct and rich in petroleum degraders and biosurfactant producers. Notably, the presence of benzene promoted the growth of specific microbial communities, including *Geobacter* species and various bacteria families such as *Arcobacteraceae, Enterobacteriaceae, Dysgonomonadaceae, Crocinitomicaceae, Oscillospiraceae, Nocardiaceae,* and *Rikenellaceae*, underscoring the niche provided by benzene for specialised benzene-degrading microorganisms. Metabolite analysis revealed that these communities were able to remove all the benzene. This is the first study, where the overlook of biosurfactant producers and petroleum degraders is provided for the bioelectrochemical system and the first study where this parameter is being analysed for the efficient degradation of benzene in the bioelectrochemical system.

Furthermore, our results demonstrate the importance of co-metabolites in MFC systems aimed at degrading petroleum compounds such as benzene. Microorganisms exposed to benzene exhibited a relatively prolonged period of adaptation to harsh environmental conditions, suggesting the need for sustained efforts in optimising the operation of the MFC for benzene degradation. Understanding the impact of co-metabolites, inoculation strategy, microbial community structure and diversity is important in terms of the practical use of MFC technology in the bioelectroremediation processes.

## Data availability statement

Original datasets are available in a publicly accessible repository: RepOD. The original contributions presented in the study are publicly available. This data can be found here: https://doi.org/10.18150/IHWMJF.

## Author contributions

NT: Formal analysis, Investigation, Methodology, Software, Visualization, Writing – original draft. JT: Formal analysis, Methodology, Software, Writing – review & editing. PM: Methodology, Writing – review & editing. GP: Conceptualization, Formal analysis, Funding acquisition, Methodology, Project administration, Resources, Supervision, Writing – original draft, Writing – review & editing.
