# Supplementary material for: The influence of benzene on the composition, diversity and performance of the anodic bacterial community in glucose-fed microbial fuel cells
Source: Front Microbiol. 2024 Jul 15;15:1384463. doi: 10.3389/fmicb.2024.1384463 (PMC11284109; doi:10.3389/fmicb.2024.1384463)
Supplement: Supplementary file 1 [file Data_Sheet_1.pdf]

frontiers

# Supplementary Material

## 1 Supplementary Figures and Tables

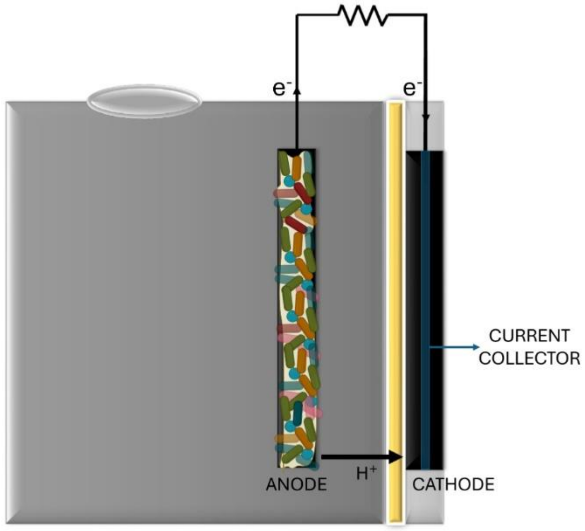


### Figure S1 MFC configuration.

### Table S1 Characteristics of qPCR primer pairs and programs.

| Primer | Sequence 5'-3' | Target gene | Amplicon size (bp) | Primer concentration (uM) | qPCR program | LOQ[1] | Reference | Amplification efficiency (samples)[2] |
|---|---|---|---|---|---|---|---|---|
| A519F | CAGCCGCCGCGGTAA | Archaeal 16S rRNA | 395 | 0.6 | 50°C 2 min, 95°C 10 min; 35 cycles: 95°C 15 s; 56°C 30 s; 72°C 30s | 1000 | [1] | 1.95 ± 0.07 |
| A915R | GTGCTCCCCCGCCAATTCCT | | | | | | | |
| B517F | GCCAGCAGCCGCGGTAA | Bacterial 16S rRNA | 530 | 0.6 | 50°C 2 min, 95°C 10 min; 35 cycles: 95°C 30 s; 60°C 45 s; 72°C 45s | 1000 | [2] | 1.85 ± 0.05 |
| B1028R | CGACARCCATGCASCACCT | | | | | | [3] | |

[1]LOQ – limit of quantification per qPCR reaction.

[2]Amplification efficiencies derived from LinRegPCR program are presented in the range of one to two, where one corresponds to no amplification (0% efficiency) and two corresponds to doubling of amplification product in each cycle (100% efficiency).

**Table S2 qPCR analysis results.**

| Sample | Substrate | Bacterial DNA copies/mL | Archaeal DNA copies/mL |
|---|---|---|---|
| MFC1A | Glucose | 3.07E+10 | 2.38E+01 |
| MFC2A | | 3.38E+10 | 1.07E+02 |
| MFC3A | | 4.03E+10 | 1.17E+02 |
| MFC4A | | 3.28E+10 | 4.11E+02 |
| MFC5A | | 7.70E+09 | 4.93E+01 |
| MFC1B | Benzene + glucose | 4.40E+10 | 1.80E+02 |
| MFC2B | | 3.85E+10 | 1.18E+03 |
| MFC3B | | 3.58E+10 | 5.62E+03 |
| MFC4B | | 6.58E+10 | 2.27E+03 |
| MFC5B | | 3.46E+10 | 6.34E+02 |

## 2 References

[1] K. Tiirik, H. Nõlvak, M. Truu, A. Peeb, M. Kõiv-Vainik, and J. Truu, "The effect of the effluent from a small-scale conventional wastewater treatment plant treating municipal wastewater on the composition and abundance of the microbial community, antibiotic resistome, and pathogens in the sediment and water of a receiving stream," Water (Switzerland), vol. 13, no. 6, Mar. 2021, doi: 10.3390/w13060865.

[2] Liu, Z.; Lozupone, C.; Hamady, M.; Bushman, F.D.; Knight, R. Short pyrosequencing reads suffice for accurate microbial community analysis. Nucleic Acids Res. 2007, 35(18), e120, doi: 10.1093/nar/gkm541.

[3] Dethlefsen, L., Huse, S., Sogin, M.L., Relman, D.A. The pervasive effects of an antibiotic on the human gut microbiota, as revealed by deep 16s rRNA sequencing. PLoS Biol. 2008, 6(11), 2383-2400, doi:10.1371/journal.pbio.0060280.
